# Determination of Possible Adulteration and Quality Assessment in Commercial Honey

**DOI:** 10.3390/foods12030523

**Published:** 2023-01-24

**Authors:** Didem P. Aykas

**Affiliations:** Department of Food Engineering, Faculty of Engineering, Adnan Menderes University, 09100 Aydin, Turkey; didem.cinkilic@adu.edu.tr

**Keywords:** honey, authentication, quality traits, FT-IR, chemometrics

## Abstract

This study aims to predict several quality traits in commercial honey samples simultaneously and to reveal possible honey adulteration using a field-deployable portable infrared spectrometer without any sample preparation. A total of one hundred and forty-seven commercial honey samples were purchased from local and online markets in Turkey and the United States of America (USA), and their soluble solids (°Brix), pH, free acidity, moisture, water activity (aw), glucose, fructose, sucrose, and hydroxymethyl furfural (HMF) contents were determined using reference methods. The HMF (*n* = 11 samples) and sucrose (*n* = 21) concentrations were higher than the regulatory limits in some tested samples. The exceeding HMF content may imply temperature abuse during storage and prolonged storing. On the other hand, high sucrose content may indicate possible adulteration with commercial sweeteners. Therefore, soft independent modeling of class analogies (SIMCA) analysis was conducted to reveal this potential sweetener adulteration in the samples, and the SIMCA model was able to identify all the flagged samples. The suggested FT-IR technique may be helpful in regulatory bodies in determining honey authenticity issues as well as assessing the quality characteristics of honey samples in a shorter period and at a lower cost.

## 1. Introduction

Honey is a naturally occurring foodstuff widely consumed for its sweetness, nutritional content, and health advantages [1]. Honey is a natural sweet substance produced by honeybees from plant nectar, secretions of living plant parts, or excretions of plant-sucking insects on living plant parts, which the bees collect, transform by combining with specific substances of their own, deposit, dehydrate, store, and leave in the honeycomb to ripen and mature [2]. Honey’s nutritional value predominantly comprises water, sugars (mainly fructose (~38%) and glucose (~31%)), and some minor compounds. These minor ingredients like proteins, enzymes, free amino acids, minerals, vitamins, organic acids, and phenolic compounds contribute to the quality and health benefits of honey [1,3,4]. These molecules give specific/individual organoleptic and nutritional features to honey and vary amongst honey due to factors such as botanical origin, geographic location, season, the honey extraction process, and storage conditions. Even in trace amounts, they are said to act as a fingerprint and are thus used to distinguish honey by origins and define their quality [1,4].

The composition of honey is tied to its botanical and geographical origin, and it can alter significantly depending on the storage period and storage conditions [3,5]. Moisture content, reducing sugars, free acids, electrical conductivity, sucrose concentration, and HMF content all impact honey’s nutritional quality, granulation, taste, and texture [6]. In addition to these compounds, phytochemical substances (i.e., phenols, sapogenin, sparteine, lunamarine, flavanone, and proanthocyanin) found in honey have an important part in determining antioxidant activity, which can be linked to honey’s anti-inflammatory, anti-carcinogenic, anti-thrombotic, and anti-atherogenic properties [6,7,8]. Because of the unique flavor and health benefits supplied by the previously mentioned chemical components in honey, it is more expensive than other sweeteners, such as cane sugar syrup, and the quality and price of honey vary greatly [9]. Unfortunately, like other high-quality nutritious and medicinal food items, with quite a high price, honey is sometimes susceptible to direct or indirect adulteration with inferior sugar syrups, resulting in the degradation of bioactive component signatures [10]. Cheaper ingredients and commercial syrups, including high fructose corn syrup, corn sugar syrup, glucose syrup, inverted sugar syrup, high fructose inulin syrup, cane sugar syrup, and rice and wheat syrups, are possible adulterants in honey [9]. Adulteration changes the chemical components, degrades the bioactive substances that are beneficial to health, and reduces the quality and value of honey [11]. When compared to a control, honey tampered with by adding sugar can, in fact, exhibit variations in several chemical and/or biological characteristics, such as enzymatic activity, electrical conductivity, and concentrations of particular chemicals (glucose, fructose, sucrose, maltose, isomaltose, HMF, proline, and ash) [1]. It might have a further detrimental impact on consumer confidence and honey producers’ income. As a result, developing precise and dependable authenticity detection systems that can also be implemented for large-scale industrial applications is critical for developing the honey business [11].

The determination of quality traits of honey and the authentication of honey has been widely studied in the literature. Honey authentication studies employed various instruments, including high-performance liquid chromatography (HPLC) [12,13], nuclear magnetic resonance (NMR), enzymatic activity, electronic tongue, stable carbon isotopic ratio mass spectrometry (SCIRA), and mass spectroscopy. The quality traits were also determined using similar analytical techniques such as HPLC, NMR, and SCIRA. However, the expense of equipment, the length of sample preparation and analysis time, and the need for a well-trained operator make it impossible for these approaches to be quickly and extensively deployed as regulatory tools.

On the other hand, vibrational spectroscopy delivers structural and fingerprint information from distinctive molecules of different substances, which provides trustworthy information for food authenticity verification. The sample preparation required for these approaches is minimal in most food industry applications, giving simplicity. Furthermore, the equipment’s ease of use eliminates the requirement for trained or specialist people. Additionally, optics and semiconductor technology advances have aided in creating portable and handheld devices. This provides in situ analysis, providing the industry and regulatory authorities with a credible and “out-of-the laboratory” tool for label verification. Even though there are several studies on the determination of the quality traits and the authenticity of honey using infrared methods in the literature, there is still a need for a comprehensive study of honey samples from various local markets and online stores.

The objective of this study was to determine the main quality traits of the honey samples collected from local markets and online stores in Turkey and the United States of America (USA) and to determine any possible economically motivated adulteration in the tested honey samples using a portable FT-IR sensor.

## 2. Materials and Methods

### 2.1. Samples

One hundred and forty-seven commercial honey samples were collected from local markets and online stores in Turkey and the USA. The samples were kept in dark and airtight containers at a refrigerator temperature until further analysis. At the time of analysis, samples were placed into a lab incubator (Precision Standard Incubator, PR205125G, Thermo Fisher Scientific, Waltham, MA, USA) that works at 45 °C for 30 min to prevent any crystallization and liquefy all the samples at the same level. However, samples were kept for another 15 min before the analysis to bring the samples to room temperature. All analyses were performed in triplicate.

### 2.2. Reference Analysis

#### 2.2.1. Sugar Analysis

Samples were prepared for the sugar analysis by mixing 1 g of the sample with 10 mL of boiling HPLC water. Samples were then vortexed for 30 s and placed in a lab incubator (Precision Standard Incubator, PR205125G, Thermo Fisher Scientific, Waltham, MA, USA) for 30 min that works at 45 °C. Samples were then vortexed for another 30 s and filtered through 0.45 μm pore nonsterile syringe filters (Phenomenex^®^, Torrance, CA, USA) into the HPLC vials.

Individual monosaccharides, including glucose and fructose, and disaccharide sucrose were quantified using High-Performance Liquid Chromatography (HPLC) (Shimadzu Scientific Instruments, Inc., Columbia, MD, USA). The HPLC system was equipped with an LC-6AD pump, SIL-20AHT autosampler, CTO-20A column oven, and a RID-10A refractive index detector. The elution of the components was realized in a stainless-steel column (RezexTM RCM-Monosaccharide Ca+ LC, Phenomenex^®^, Torrance, CA, USA). The column was 7.8 mm ID × 300 mm, placed in an even working at 80 °C, and an HPLC grade water was used as a mobile phase with 1 mL/min flow rate isocratically. The run was completed in 20 min for each sample. Attained chromatograms were evaluated by LC Solutions software version 3.0 (Shimadzu, Columbia, MD, USA). The individual sugar contents were assessed by generating an external calibration curve. The calibration curve was made by running the known concentration (1.56 and 50 mg/mL) standards (glucose and fructose) (Fisher Scientific, Fair Lawn, NJ, USA) using HPLC under the same conditions as the honey samples.

#### 2.2.2. Soluble Solid Content (°Brix)

The soluble solid content of the honey samples was determined by placing the honey samples (~0.3 g) directly onto the prism of the temperature-controlled refractometer (RX 5000i ATAGO, Bellevue, WA, USA). The equipment was zeroed by using distilled water before the analysis. The readings were conducted at 21 °C.

#### 2.2.3. Water Activity

The water activity of the honey samples was measured using a water activity meter (aw) (AQUALAB 3, Decagon, Pullman, WA, USA) at 25 °C, and ~5 g of a honey sample was placed into the container for each analysis. Prior to the analysis, the instrument calibration was performed using saturated salt solutions with a range of aw (0.25 and 0.75).

#### 2.2.4. pH Measurement

The pH values of the samples were determined using a pH meter (Mettler-Toledo, Inc., Columbus, OH, USA) at room temperature. Samples were prepared by diluting them with distilled water (10% *w*/*v* dilution).

#### 2.2.5. Moisture Content

The moisture content of honey samples was evaluated using the AOAC Official Method (969.38) [14]. According to this method, a honey sample was placed onto the surface of the digital refractometer, and the measurements were conducted at 20 °C. Then the refractive index reading was converted to the moisture content in percentage using the Chataway Table with Wedmore’s corrections [15].

#### 2.2.6. Hydroxymethyl Furfural (HMF)

The HMF content of the honey samples was determined using the method provided by the International Honey Commission [16], with slight modifications. Thus, approximately 1 g of honey sample was diluted with 5 mL of HPLC-grade water into the volumetric flask and, after dissolving the sample well, filtered through 0.45 μm pore nonsterile syringe filters (Phenomenex^®^, Torrance, CA, USA) into the amber-colored HPLC vials, and 20 μL sample was injected to the column. The elution of the samples was carried out at an HPLC (1100 Series, Agilent Technologies, Santa Clara, CA, USA) composed of a G1311A quaternary pump, a G1322A degasser, a G1313 ALS autosampler, a G1316A column compartment, and a G1315B diode array detector. The column was a Symmetry C18 (3.5 μm, 150 × 4.6 mm, Waters Corp., Milford, MA, USA), and the separation was at the room temperature, and the mobile phase (water: methanol, 90:10 (*v*/*v*)) flow rate was 1.0 mL/min isocratically. The HMF content was quantified at 285 nm.

#### 2.2.7. Free Acidity

The free acidity of the honey samples was determined using the International Honey Commission method [16]. Accordingly, 10 g of honey samples were dissolved in 75 mL distilled water in a 250 mL glass beaker and titrated using standardized 0.1M NaOH until reaching pH 8.3.

### 2.3. Spectral Analysis

The mid-infrared spectra of the samples were collected using a portable Fourier transform mid-infrared (FT-IR) spectroscopy equipped with a triple-reflection diamond Attenuated Total Reflectance (ATR) crystal (4500, Agilent Technologies, Santa Clara, CA, USA). The FT-IR sensor had a Zinc Selenide beam splitter, thermoelectrically cooled deuterated triglycine sulfate (dTGS) detector, low-powered solid-state laser, and wire-wound element infrared source. Samples’ spectra were acquired at room temperature, over a range of 4000–700 cm^−1^, with a resolution of 4 cm^−1^, and 64 co-scans were co-added to improve the signal-to-noise ratio. A total of 75 µL of the sample was directly deposited onto the crystal and to diminish the possible effect of environmental factors background spectrum was collected before every spectral measurement. Spectral data were displayed in absorbance and viewed and recorded using Agilent MicroLab PC software (Agilent, Santa Clara, CA, USA).

### 2.4. Multivariate Data Analysis

Collected spectra were evaluated using multivariate data analysis software (Pirouette^®^, v4.5, Infometrix Inc., Bothell, WA, USA).

#### 2.4.1. Partial Least Square Regression Analysis

Quality traits for the honey samples were predicted by correlating the reference analysis results of each parameter with the spectral data set. The partial least square regression (PLSR) methodology was used for the correlation analysis. PLSR is one of the commonly used data compression techniques that determine a set of factors that captures the maximum correlation between predictor (i.e., spectra from infrared sensors) and predicted (i.e., concentration results from the traditional reference analysis), and also explains the maximum variance related with these variables [17]. Before the analysis, the data set was randomly divided into two subgroups (training/calibration and external validation sets). The training set comprised 80% of the entire data set, while the external validation had the remaining 20% of the samples. The primary purpose of subgrouping the data set was to evaluate the prediction performance of the generated training model. If the external validation set provided similar statistical performance to the training set, the generated training model was considered robust. Besides the external validation, the model was also internally validated through the cross-validation (leave-one-out) approach. This approach also facilitates choosing the optimal number of factors (latent variables—LVs). In this process, if the data set consists of *n* number of samples, one sample leaves out each time, and the model is trained for the remaining samples (*n*-1). Then, that model was tested with the left-out sample, which was applied to the remaining samples one by one. As a result, it happens *n* times until no sample is left without testing. Even though this process is computationally expensive, it reduces the variance and helps to find the optimal number of LVs. Selecting the optimal number of LVs is the key to building an accurate and reliable PLSR model since choosing a smaller number of LVs could result in not including all the relevant information/variance (underfitting) while choosing many numbers of LVs could result in including unnecessary information (noise) into the model (overfitting) [18].

Residual prediction deviation (RPD) and range error ratio (RER) are two different concepts that can be used to further evaluate the robustness and accuracy of the generated PLSR models. Therefore, the RPD and RER values were also used to evaluate generated PLSR models besides internal and external validation. The RPD is a unitless value, and it can be calculated by diving the standard deviation of the reference data (i.e., data from HPLC-RID, aw meter, refractometer, etc.) in the calibration set to the standard error of prediction (SEP) of the external validation set. Models with an RPD value of 2.0–2.4 can only be used for rough screening purposes, 2.5–2.9 are acceptable for screening, 3.0–3.4 can be used for quality control purposes, 3.5–4.0 are applicable for process control and higher than 4.1 are practicable for all type of applications [19]. RER, on the other hand, is another unitless measure and can be calculated from the ratio between the external validation set’s reference data range to the SEP. The RER value is typically higher than the RPD value, and the model’s accuracy rises as the RPD or RER value is raised [20]. As a result, the models with higher than 4.0 RER, 10.0 RER, and 15.0 RER can be utilized for screening, quality control, and quantification, respectively. Furthermore, the number of latent variables (LVs), scores, loadings, standard error of cross-validation (SECV), the correlation coefficient of cross-validation (R_CV_), and outlier diagnostics were used to assess to determine the performance of the generated regression models.

#### 2.4.2. SIMCA

Soft independent modeling of class analogies (SIMCA) is a principal component analysis-based supervised classification approach. The word “soft” is used since the model does not require the sample to assign to any classes, and the sample can assign to a class, numerous classes, or none at all [21]. SIMCA, as the name implies, creates independent models for each class by performing a principal component analysis (PCA) on each class individually. Additionally, the number of principal components (PCs) is selected independently for each group. SIMCA takes preexisting knowledge about class memberships (pure/authentic vs. contaminated) and separates and creates each class independently using PCA, maintaining just the relevant components [22]. Samples are grouped in a class according to their Euclidian distance from its PC space, and this Euclidean distance does not exceed a critical distance, which is based on F-distribution and calculated as confidence intervals (95 or 99%). Then, for the new samples, residuals (errors) take into consideration (assigned to that class if its residual distance is below the statistical limit for that class), and that sample can be grouped as similar or dissimilar from those determined groups [23,24]. SIMCA provides various diagnostic tools to help users better understand the findings, including the distance between classes (also known as interclass distances (ICDs)), residuals between classes, modeling power, and discriminating power. ICD is a unitless metric that identifies similarities between two separate classes; hence, if the ICD between two classes is more than three, those two classes are typically acknowledged as substantially different in the acceptable confidence intervals (95% for this research). On the other hand, the discriminating power plot visualizes the spectral bands responsible for the sample categorization.

Before commencing the SIMCA analysis, the data were separated into a training set and an external validation set, and the SIMCA model was built using the training set. On the other hand, the constructed SIMCA model’s performance was evaluated using the external validation set. The external validation set was made up of previously unseen data from the training set. The SIMCA models’ performances were also evaluated using misclassification, discriminating power, ICD, class projections, sensitivity, specificity, accuracy, and precision.

## 3. Results

### 3.1. Characterization of Honey Samples

Reference analysis results for tested quality traits for the analyzed honey samples are summarized in Table 1, and the findings conform with the literature [6,25,26,27,28,29,30,31,32,33,34]. The soluble solids (°Brix) concentration of the tested samples ranged from 72.4 to 82.7, with an average of 78.7 (Table 1), which was similar to other studies [6,25,28,29,35]. Even though Geana et al., 2020 [30] and Terrab et al., 2004 [36] reported similar soluble solid content for most of their tested samples, honeydew honey (85.36 °Brix), sunflower honey (83.62 °Brix), and some Spanish thyme honey were reported having higher °Brix values.

pH values of the honey samples ranged between 3.73 and 4.61 with an average of 4.02, and the findings were similar to the literature [6,25,26,27,28,29,30,31,32,33,35,37]. The botanical origin of the plant, the pH of the nectar, the association of the soil or plants, and the concentration of various acids and minerals, such as calcium, sodium, potassium, and other ash elements, all affect the pH value of the honey [38]. The free acidity content of the tested samples was ranging in between 8.17 and 38.9 meq/kg (average of 27.9 meq/kg) (Table 1). According to the Council of the European Union, honey cannot have more than 50 meq/kg, and all our tested samples complied with this limit.

As it affects honey’s viscosity, specific gravity, maturity, crystallization, taste, preservation, shelf life, and palatability, water content is one of its most crucial properties, and it relies on several variables, including the kind of bees, the type of flowers used, the timing of honey collection, the level of maturity attained in the hive, and environmental circumstances [38]. The potential of honey to maintain stability and resist deterioration by yeast fermentation is determined by its moisture content; the higher the moisture, the more likely it is that honey will ferment when stored [16]. High moisture levels can cause the honey to crystallize and encourage the growth of osmophilic yeast that cause fermentation, which negatively affects the product’s sensory qualities and nutritional qualities and shortens its shelf life [38,39]. According to the Council of the European Union, the moisture content limit for honey is 20%, and all our tested samples complied with this limit, ranging from 13.6 to 19.7% (Table 1). In the literature, Terrab and others (2004) [36] also found similar results for moisture content in Spanish thyme honey, and Oroian and others (2017) [6] found similar content in Romanian honey. On the other hand, Can and others (2015) [27] determined slightly higher moisture content in some of the Turkish honey (Heather—20.86% and Acacia—20.8% moisture). Furthermore, the water activity of the tested samples ranged from 0.45 to 0.62 with an average of 0.53 (Table 1) by having the average value below the crucial value of 0.6 aw for osmophilic yeast growth that causes honey fermentation [39], guaranteeing a reasonably extended shelf life for the honey samples.

The majority of honey’s dry weight, or around 95% of it, is made up of sugars. The hydrolysis of the disaccharide sucrose yields the monosaccharide hexoses fructose and glucose, which are the most prevalent sugars in honey [40]. According to the Codex Alimentarius and the Council of the European Union, pure honey should have more than 60 g of total glucose and fructose concentration per 100 g of honey, and the sucrose concentration should not exceed 5 g per 100 g of honey [41,42]. In this study, fructose is present in greater concentrations than glucose in the majority of the samples. The glucose and fructose concentrations in the analyzed samples ranged from 15.3 to 39.2% and 31.2 to 48.4 %, respectively (Table 1). The sucrose concentration in the samples was determined at an average of 2.1% (Table 1) which complies with the studies in the literature [25,30,34]. However, a total of 21 samples’ sucrose concentrations were above the 5 g/100 g (%) limit; therefore, those samples were flagged as being suspicious in this study.

HMF is a furanic decomposition product of the fructose compound that is typically absent or just minimally present in fresh, unprocessed honey [40]. HMF is typically utilized as a sign of honey’s quality and freshness since it can develop in honey as a result of overheating or during long-term storage [1,43]. With the exception of honey from tropical regions, the EU’s regulations suggest that its content should not be more than 40 mg/kg [1]. The HMF contents of the tested samples were between 17.6 and 86.9 mg/kg with an average of 34.6 mg/kg, even though some samples (*n* = 11) went beyond the advised standard limits (40 mg/kg), possibly due to improper storage conditions or prolonged shelf-life. The outcomes were consistent with those of previously published research [35].

### 3.2. Spectral Characteristics of the Samples

**Figure 1:**
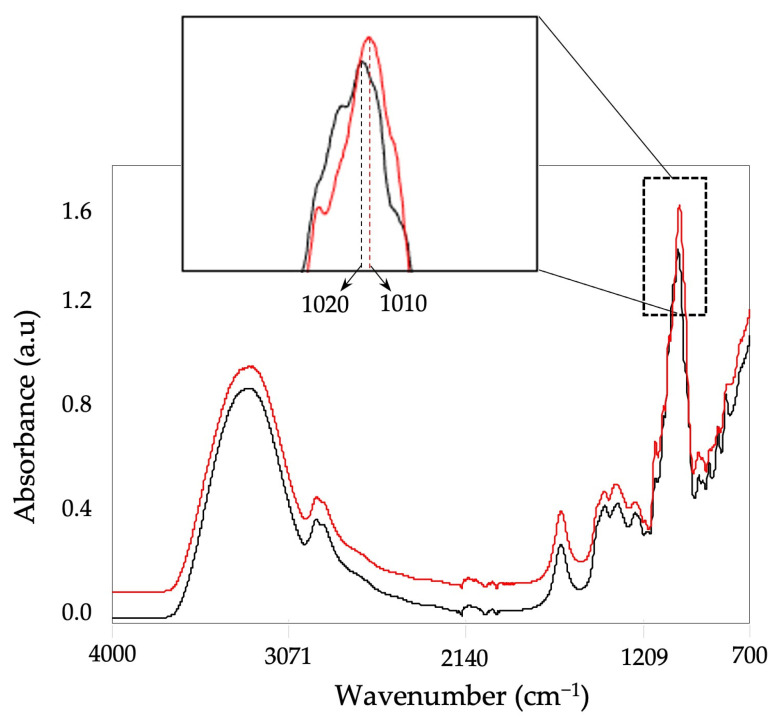
Average raw spectra of the pure honey samples and suspicious samples with high sucrose concentration (>5%). The spectra were collected using a portable FT-IR sensor at a range of 4000–700 cm^−1^. The red line demonstrates the suspicious sample with high sucrose content, the black line is the pure honey.

Figure 1 displays averaged raw FT-IR absorption spectra of all pure and potentially contaminated honey samples together with their corresponding band allocations for various functional groups. Overall, all the pure honey samples (sucrose concentration < 5%) showed a similar spectral pattern throughout the 4000–700 cm^−1^ range. Even though the suspicious samples (having more than 5% sucrose) showed a similar pattern with the pure samples, especially at the 1020–1010 cm^−1^ range, they provided a distinction from the pure samples (Figure 1).

The bands centered at 3285 cm^−1^ and 1637 cm^−1^ were associated with O-H stretching and O-H deformation, respectively [44]. The absorbance band at 2930 cm^−1^ related to C-H stretching of the CH_2_ group in carbohydrates with a minor contribution from -NH_3_^+^ of free amino acids [45,46,47]. The region between 1500 and 750 cm^−1^ is associated with the main components of honey, mainly sugars and organic acids [47]. Specifically, the bands at 1411 and 1321 cm^−1^ are associated with the O-H bending of C-OH groups. Additionally, the C-H bending of alkenes provided some absorption in the former wavenumber [45,47]. The 1110 cm^−1^ band is linked to the C-O stretching of the C-O-C linkage, which might be related to the glycosidic bond in sucrose [45,47]. The C-O stretching in the C-OH group and the C-C stretch in the carbohydrate structure provided absorbance at 1043 cm^−1^ and 1254 cm^−1^ [47]. The band at 918 cm^−1^ is associated with the carbohydrate’s C-H bending [47]. Carbohydrates may generally be characterized as being in the 800–1200 cm^−1^ range, whereas organic and amino acids reside in the 1200–1800 cm^−1^ region [45].

The remarkable band for the pure honey samples at 1020 cm^−1^ is related to the C–O and C–H stretching [44]. On the other hand, all the suspicious samples provided a shift at this wavenumber and provided the absorption at 1010 cm^−1^ (Figure 1). Similarly, Cardenas-Escudero and others [44] reported the same pattern with pure honey and rice syrup.

### 3.3. Validated PLSR Models

Using the infrared spectra from the portable FT-IR sensor and the reference analysis results, quantitative prediction models for nine quality traits of honey samples were generated. Outliers and samples with large leverages were removed from the sample set before the calibration and external validation models were built, and the sample set was then randomly split into these two groups. Specific wavenumbers from the FT-IR spectral region were chosen specifically for each quality trait to get the best model performances and exclude irrelevant, noisy, and unreliable variables (wavenumbers). Most of the spectroscopic studies in the literature evaluate the quality of the calibration models in terms of linearity and accuracy [48]. The linearity also indicates, by the coefficient of determination (R^2^), the degree of variability of the reference data that the regression equation can explain. The standard error of cross-validation (SECV) reflects the variability in the difference between predicted and reference values when the equation was constructed using the cross-validated calibration data set, which can be used to estimate the accuracy of the generated model [48]. Accordingly, Models with a high R^2^ and a low SECV show a good fit to the calibration data. The SECV indicates the degree of error to be anticipated when the generated models are used to forecast unknown samples similar to the generated model’s data [49].

Table 2 demonstrates the statistical performances of the generated PLSR models of calibration and external validation, besides the number of samples used in each model and the range of values. In order to create the FT-IR calibration models, cross-validation (leave-one-out approach) revealed three to six components (Table 2), explaining between 92 and 99% of the total variance, depending on the quality trait. Models for honey quality traits produced strong coefficients of determination (≥0.92) (Table 2), suggesting that the data firmly grouped along the regression line for all quality parameters. Furthermore, the generated calibration models provided low prediction errors (SECV) to predict the soluble solids (0.51 °Brix), pH (0.06), free acidity (2.81 meq/kg), moisture (0.38%), water activity (0.03), glucose (2.12%), fructose (2.58%), sucrose (0.19%), and HMF (4.17 mg/kg).

In order to validate the models, a separate set of samples (20% of the total data set) was employed. Models were found to have similar R^2^_CV_ and R^2^_Pre_ values and similar SECV and SEP values for all quality parameters, demonstrating the models’ resilience under real-world circumstances (Table 2). The prediction models performed similarly to those previously reported to predict honey quality traits [25]. Furthermore, the performances of the generated models were further evaluated using the RPD and RER values. Calculated RPD and RER values suggest that the pH, free acidity, moisture, water activity, glucose, fructose, and sucrose models can be used for quality control applications (3.4 > RPD > 3 and/or RER > 10), while soluble solids and HMF models can be used for process control purposes (RPD > 3.4 and RER > 15) (Table 2).

### 3.4. SIMCA Classification Models

The regulation from Codex Alimentarius [41] states that the sucrose level of genuine honey should not be higher than 5/100 g honey (%), and the sum of glucose and fructose concentration in honey should not be less than 60/100 g honey (%). According to the HPLC-RID sugar analysis, twenty-one out of hundred and forty-seven samples were determined to have unusual sugar profiles by having a high sucrose content (>5%). On the other hand, the rest of the honey samples provided compatible sucrose concentrations with the regulations.

**Figure 2:**
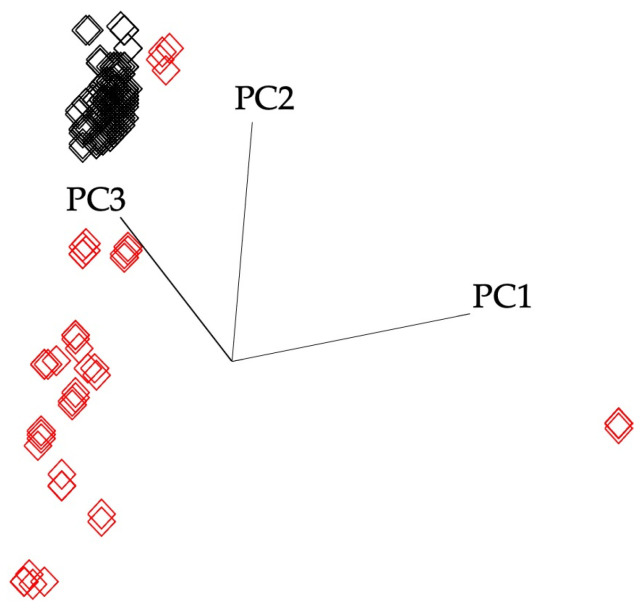
Soft independent modeling of class analogy (SIMCA) 3D projection plot of spectral data for honey samples collected by portable FT-IR sensor. The red squares show suspicious honey samples, whereas the black ones indicate the pure honey samples.

**Figure 3:**
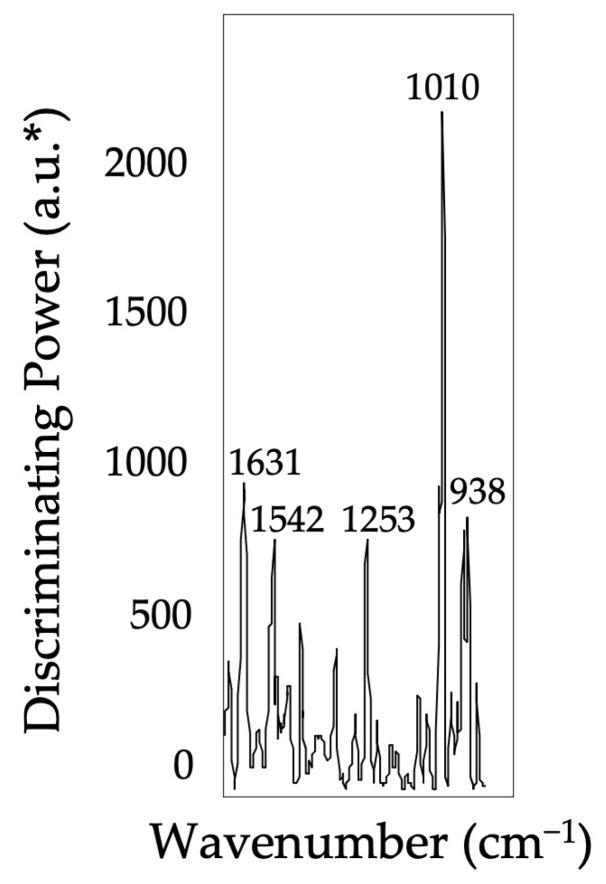
SIMCA discriminating plot based on the FT-IR spectra of pure and suspicious honey samples using the portable FT-IR sensor, showing bands and regions responsible for the class separation. * arbitrary units.

Pure honey and dubious samples’ spectra were investigated/distinguished using a supervised pattern recognition technique, SIMCA, to extract meaningful spectral information from the highly complex spectral data. Prior to the analysis, the pure (*n* = 126) and the suspicious (*n* = 21) samples were randomly split into the training/calibration (80%) and the external validation (the remaining 20%) sets. Accordingly, the training set consist of 101 pure and 17 suspicious samples, and all the pure samples were given the same class label (#1), whereas samples of suspicious samples were given a different class label (#2). Four factors were included to generate the training model, which explained 99.3% of the variations, and the training set projection graphs were displayed in Figure 2. The interclass distance (ICD) of the pure honey and suspicious samples’ classes was determined to be 13.01. It can be concluded that these two classes (pure honey #1 and suspicious samples #2) were significantly different than each other since the ICD was higher than three. Furthermore, the generated and cross-validated SIMCA model provided zero misclassification that suggests the model minimizes the over-fitting. The key bands associated with the greatest variance and accountable for the class separations were shown in the discriminating power plot of the SIMCA training models along the chosen spectral ranges (Figure 3). The region between 1650 and 920 cm^−1^ was used to discriminate pure honey samples from the suspicious ones using the FT-IR sensor. The C-O and C-H stretching, which is strongly connected to the intensity differences of bands centered at 1010 cm^−1^, accounted for the majority of the model variation.

The SIMCA models’ ability to predict future (new/unseen) samples were assessed using a separate validation set. The external validation set (*n* = 25 authentic honey samples, *n* = 4 suspicious samples) revealed the generated training model was robust to predict new samples since all the performance statistics provided 100% specificity, sensitivity, accuracy, and precision.

## 4. Conclusions

The present study investigated the application of a portable FT-IR spectroscopy combination with chemometric analysis to predict the main quality attributes of the commercial honey samples (*n* = 147) collected from local markets and online stores in Turkey and the USA. Furthermore, a total of 21 samples were determined as having remarkably high levels of sucrose concentration. Using the same spectra, samples with a high sucrose content were discriminated from the pure honey samples. This research was able to prove that the fingerprinting capabilities of the mid-infrared region offer a unique signature profile that, with the use of supervised pattern recognition techniques, permitted the identification of chemical differences in tampered ingredients. The potential profits and trading advantages from mislabeling prejudice the interests of both consumers and honest manufacturers, and the data of honey samples collected from commercial markets, strongly supports that the portable FT-IR instrument presents great potential for efficient in situ surveillance of pure honey from honey mixtures and cheaper alternatives.

## Figures and Tables

**Table 1:** Reference concentration levels for the measured quality traits in honey samples.

Parameter	Minimum	Maximum	Average	STDEV ^b^
Soluble solids (°Brix)	72.4	82.7	78.7	3.2
pH	3.73	4.61	4.02	0.2
Free acidity (meq/kg)	8.71	38.9	27.9	6.8
Moisture (%)	13.6	19.7	15.8	0.9
Water activity	0.45	0.62	0.53	0.04
Glucose (%)	15.3	39.2	30.2	5.1
Fructose (%)	31.2	48.4	39.4	2.7
Sucrose (%)	0.3	12.6	2.1	2.1
HMF (mg/kg) ^a^	17.6	86.9	34.6	7.8

^a^ Hydroxymethyl furfural, ^b^ standard deviation.

**Table 2:** Performance statistics of calibration and external validation models developed by using portable FT-IR sensor.

	Calibration Model					External Validation Model					
Parameter	Range	N ^b^	Factor	SECV ^c^	R^2^_CV_ ^d^	Range	N ^e^	SEP ^f^	R^2^_Pre_ ^g^	RPD ^h^	RER ^i^
Soluble solids (°Brix)	72.4–82.7	118	3	0.51	0.98	72.7–81.6	29	0.47	0.98	5.6	19.8
pH	3.73–4.61	116	5	0.06	0.95	3.75–4.55	29	0.06	0.94	4.1	10.2
Free acidity (meq/kg)	8.71–38.9	117	5	2.81	0.96	9.48–32.1	29	2.93	0.97	5.3	12.8
Moisture (%)	13.6–19.7	117	4	0.38	0.95	14.1–17.6	29	0.42	0.94	3.2	15.1
Water activity	0.45–0.62	117	6	0.03	0.92	9.47–0.59	29	0.03	0.94	3.8	14.9
Glucose (%)	15.3–39.2	117	4	2.12	0.96	18.2–35.4	29	2.68	0.95	4.6	11.3
Fructose (%)	31.2–48.4	117	4	2.58	0.96	32.4–45.1	29	3.01	0.96	4.9	10.9
Sucrose (%)	0.3–12.6	118	3	0.19	0.94	0.35–10.4	29	0.14	0.94	3.2	14.1
HMF (mg/kg) ^a^	17.6–86.9	116	4	4.17	0.96	19.6–63.2	29	3.91	0.97	5.7	17.5

^a^ Hydroxymethyl furfural, ^b^ number of samples used in calibration models, ^c^ standard error of cross-validation, ^d^ coefficient of determination of the calibration, ^e^ number of samples used in the external validation models, ^f^ standard error of prediction, ^g^ coefficient of determination of the external validation model, ^h^ residual predictive deviation, and ^i^ range error ratio.

## Data Availability

Data is contained within the article.
